# The impact of preoperative biliary drainage on postoperative outcomes in patients with malignant obstructive jaundice: a retrospective analysis of 290 consecutive cases at a single medical center

**DOI:** 10.1186/s12957-021-02476-z

**Published:** 2022-01-06

**Authors:** Zhihui Gao, Jie Wang, Sheng Shen, Xiaobo Bo, Tao Suo, Xiaoling Ni, Han Liu, Lihong Huang, Houbao Liu

**Affiliations:** 1 Department of General Surgery, Zhongshan Hospital, Fudan University, 180 Fenglin Road, Shanghai, 200032 China; 2 Department of Biostatistics, Zhongshan Hospital, Fudan University, 180 Fenglin Road, Shanghai, 200032 China

**Keywords:** Preoperative biliary drainage, Obstructive jaundice, Cholangiocarcinoma

## Abstract

**Background:**

The efficacy of preoperative biliary drainage (PBD) has been debated for several decades, and yet indications for PBD remain controversial. The aim of this study was to compare the postoperative morbidity and mortality in patients with malignant obstructive jaundice undergoing direct surgery versus surgery with PBD.

**Methods:**

All consecutive patients with malignant obstructive jaundice who underwent radical resection between June 2017 and December 2019 at Zhongshan Hospital were analyzed retrospectively. The study population was divided into two groups: PBD group (PG) and direct surgery group (DG). The subgroups were chosen based on the site of obstruction. Perioperative indicators and postoperative complications were compared and analyzed.

**Results:**

A total of 290 patients were analyzed. Postoperative complications occurred in 134 patients (46.4%). Patients in the PG group had a lower overall rate of postoperative complications compared with the DG group, with perioperative total bilirubin (TB) identified as an independent risk factor in multivariate analysis (hazard ratio = 1.004; 95% confidence interval 1.001–1.007; *P* = 0.017). Subgroup analysis showed that PBD reduced the complication rate in patients with proximal obstruction. In the proximal-obstruction subgroup, a preoperative TB level > 162 μmol/L predicted postoperative complications.

**Conclusions:**

PBD may reduce the overall rate of postoperative complications among patients with proximal malignant obstructive jaundice.

**Trial registration:**

ClinicalTrials.gov, 2018ZSLC 24. Registered May 17, 2018, https://clinicaltrials.gov/.

## Introduction

Biliary obstruction is commonly the first presentation in several cancers affecting the region from the perihilar bile duct to the pancreatic head, including perihilar cholangiocarcinoma (pCCA), distal cholangiocarcinoma (dCCA), ampullary carcinoma, and pancreatic ductal adenocarcinoma (PDAC) [1, 2]. Patients with intrahepatic cholangiocarcinoma (iCCA), gallbladder carcinoma (GBC), or other tumors invading the extrahepatic bile duct may also present with biliary obstruction as the first symptom [3–5]. However, these types of tumors are distinct in their presentation, natural history, and approach to diagnosis and management [6]. Surgical resection is the only well-established option that provides the best chance for long-term survival. However, the procedure is very challenging [7, 8]. Prolonged and progressive obstructive jaundice induces fatigue, malnutrition, bile stasis, cholangitis, and endotoxemia, and is associated with hepatic dysfunction, coagulopathy, infections, anastomotic leakage, and delayed recovery after the surgical operation [6, 9, 10]. Preoperative biliary drainage (PBD) procedures have been introduced to alleviate the negative effects of biliary obstruction. Nonetheless, PBD is an invasive treatment and carries a risk of procedure-related complications. Although it has theoretical value, routine PBD does not improve perioperative outcomes in patients with jaundice. Indications for PBD in malignant obstructive jaundice are still debated [11, 12].

In China, several large medical centers have attempted different kinds of perioperative management to improve resectability and curability, and to reduce surgical mortality and morbidity rates for radical resections. Advances in surgical techniques have enabled resection of locally advanced tumors. Three-dimensional printing technology provides certain guiding significance in the preoperative evaluation of accurate resection, and resection and reconstruction of the portal vein and hepatic artery are increasingly performed. PBD may have played a substantial role in these possible improvements, but there is no widely accepted standard.

Therefore, the present study aimed to assess the impact of PBD on postoperative outcomes and to identify in which cases PBD should be recommended.

## Methods

### Patients and study design

Data on the patients that underwent radical resection due to malignant biliary obstruction at Zhongshan Hospital Fudan University, Shanghai, China, between June 2017 and December 2019, were retrieved from the Hospital Information System and retrospectively analyzed. Only patients who achieved preoperative total bilirubin > 51 μmol/L were included. They either underwent radical resection or received PBD preoperatively. Only cases with full medical records wherein PBD was performed at our institution were included. The full medical records comprised initial imaging data, PBD and surgical operation records, hospitalization records, and perioperative laboratory testing results (within 3 days before operation, on the first, third, and seventh days after PBD or operation, and once a week after operation). Demographic and clinical data, including age, sex, the American Society of Anesthesiologists (ASA) score, body–mass index (BMI), medical history, histopathological diagnosis, preoperative physical examinations, laboratory tests, chest X-ray imaging, and computed tomography or magnetic resonance imaging of the abdomen, were collected. Informed written consent was obtained from each participating patient. A radical resection was considered complete if the entire gross tumor volume was removed with negative resection margins (R0 resection), while an incomplete resection was defined as the presence of a microscopic tumor in the surgical resection margin (R1 resection). Palliative resection was defined as the presence of any gross residual tumors (R2 resection), and such cases were excluded from this study. Antibiotic prophylaxis was administered and continued until at least postoperative day 5. All patients were treated with the second-generation cephalosporin intravenously ½ to 1 h prior to the start of the surgery. For lengthy procedures (e.g., ≥ 3 h) or if the amount of bleeding exceeded 1500 mL, additional dose was administered intravenously during surgery (administration modified depending on the duration of the operative procedure). Finally, 290 patients were enrolled in the study, of whom 159 had PBD before the resection (“PBD group”; PG) and 131 underwent an early surgical resection without PBD (“direct surgery group”; DG). The study protocol was approved by the ethics committees of Zhongshan Hospital and Fudan University. The methods were used in accordance with the approved guidelines. All postoperative complications were scored and classified using Common Terminology Criteria for Adverse Events 5.0. We assessed study quality using STROBE guidelines.

### The PBD procedure

According to the clinical practice guidelines of Zhongshan Hospital, patients with jaundice with a total bilirubin level > 200 μmol/L or with malnutrition and cholangitis were treated with PBD as a “bridge therapy” before a radical surgical resection was attempted. PBD types included percutaneous transhepatic biliary drainage (PTBD), endoscopic nasobiliary drainage (ENBD), and endoscopic biliary stenting (EBS). In cases of failure, PTBD is usually carried out as a remedial treatment. Some patients treated had been referred from low-volume centers or other hospitals where PBD had already been performed as the first intervention. For these reasons, there were no standardized indications for jaundice palliation during the study period.

The definitions of radical resections are as follows. Radical resections for proximal obstruction, mainly in pCCA and gallbladder carcinoma, were normally hepatectomies, while those for distal obstructions, mainly in biliary carcinoma, ampullary carcinoma, and PDAC, were normally pancreatoduodenectomies. Standard lymph node dissection was also performed. All the patients received intraoperative antibiotic prophylaxis.

### Statistics

The data were analyzed using the SPSS software version 25.0 (IBM Corp., Armonk, NY, USA). Categorical data are presented as percentages, and frequencies were compared by the *χ*^2^ test or McNemar’s test, as appropriate. Continuous variables were analyzed for normality using the Kolmogorov-Smirnov test. Normally distributed continuous variables are presented as means and standard deviations (SD), and the significance of pairwise differences was assessed by the independent-sample or dependent-sample *t* test, as appropriate. Non-normally distributed continuous variables are presented as medians and interquartile ranges, and the significance of their differences was evaluated by the Mann–Whitney *U* test or Wilcoxon signed-rank test, as appropriate. To identify the best cutoff of preoperative total bilirubin (TB) for distinguishing patients with postoperative complication(s), a receiver-operating characteristic (ROC) were generated, and the area under the ROC curve (AUC), sensitivity, and specificity were used to investigate the validity of TB diagnostic models. Differences with a *P* value < 0.05 were considered statistically significant.

## Results

A total of 290 consecutive patients with malignant obstructive jaundice who underwent a radical resection at Zhongshan Hospital between June 2017 and December 2019 were included in the study. Among these, 159 (54.8%) patients had received PBD (PG group) before the resection, and 131 (45.2%) had undergone a radical resection directly after admission (DG group). In PG, for 5 (3.1%), and 149 (93.7%) patients, EBS, ENBD, and PTBD were chosen as the initial PBD, respectively. Eight patients (5.0% of the 159) needed second PBD, which was PTBD in all cases. The frequency of the second PBD was not significantly different among the three subgroups (*P* = 0.082; Table 1). Radical resections included pancreaticoduodenectomy, radical resection for pCCA, and other procedures, according to the tumor site.

**Table 1:** The frequency of a second PBD in group PG

	Total	EBS	ENBD	PTBD	Statistic	*P* value
First PBD	159 (100%)	5 (3.1%)	5 (3.1%)	149 (93.7%)	*χ*^2^ = 5.665	0.082*
Second PBD	8 (5.0%)	1 (20.0%)	1 (20.0%)	6 (4.0%)		

*Fisher’s exact test in Monte Carlo mode

### Patient characteristics

Baseline characteristics of the patients with malignant obstructive jaundice in groups DG and PG are described in Table 2. The baseline characteristics (on admission), including age, sex, and BMI, were not significantly different between the two groups. ASA scores were significantly different between the two groups: the PG group had a higher ASA score III–IV (*P* < 0.001). One hundred seventy-two (59.1%) patients had proximal biliary obstruction, and 119 (40.9%) had distal biliary obstruction. The site of obstruction did not significantly affect the choice of PBD (*P* = 0.301). Final histopathological diagnoses were as follows: four iCCAs and 14 GBCs with invasion of an extrahepatic bile duct, 153 pCCAs, 51 distal biliary carcinomas, 47 ampullary carcinomas, 20 PDACs, and 1 hepatocellular carcinoma with invasion of an extrahepatic bile duct. The prevalence rates of these diagnoses were not significantly different between the groups, DG and PG (*P* = 0.301).

**Table 2:** Demographic data on the patients under study

Characteristics		Total (*n* = 290)	DG (*n* = 131)	PG (*n* = 159)	Statistic	*P* value
Age (years)	Mean ± SD (range)	62.21 ± 9.41 (24‑80)	62.21 ± 9.60 (24‑80)	62.2 ± 9.30 (28‑80)	*t* = 0.007	0.994
	≥ 70, *n* (%)	63 (21.72%)	29 (22.14%)	34 (21.38%)	*χ*^2^ = 0.024	0.877
	< 70, *n* (%)	227 (78.28%)	102 (77.86%)	125 (78.62%)		
Sex	Male, *n* (%)	185 (63.79%)	82 (62.60%)	103 (64.78%)	*χ*^2^ = 0.148	0.700
	Female, *n* (%)	105 (36.21%)	49 (37.40%)	56 (35.22%)		
BMI (kg/m^2^)	Mean ± SD	22.72 ± 3.02	22.52 ± 2.65	22.86 ± 3.25	*t* = 0.862	0.389
ASA score	I	22 (7.59%)	10 (7.63%)	12 (7.55%)	*χ*^2^ = 17.810	**< 0.001**
	II	237 (81.72%)	118 (90.08%)	119 (74.84%)		
	III‑IV	31 (10.69%)	3 (2.29%)	28 (17.61%)		
Medical history	Hepatolithiasis	12	2	10	*χ*^2^ = 1.752	0.636*
	Cholecystolithiasis	29	11	18		
	HBP	50	17	33		
	Diabetes	26	8	18		
Obstruction site	Proximal	172	82	90	*χ*^2^ = 1.068	0.301
	Distal	118	49	69		
Histopathological diagnosis	iCCA	4	3	1	*χ*^2^ = 4.187	0.664*
	GBC	14	8	6		
	pCCA	153	71	82		
	Biliary carcinoma	51	21	30		
	Ampullary carcinoma	47	21	26		
	PDAC	20	7	13		
	Hepatocellular carcinoma	1	0	1		

*HBP* high blood pressure

*Fisher’s exact test in Monte Carlo mode

### Laboratory testing results

A comparison of these data between the time of initial diagnosis and the time of perioperative testing is presented in Table 3. Within the DG group, initial laboratory test results, including hemoglobin (Hb) and ALB (albumin), were significantly higher compared with the perioperative test (*P* < 0.001 and *P =* 0.016). ALT and AST levels also decreased significantly (*P* < 0.001 and *P =* 0.005, respectively), while TB and direct bilirubin (DB) levels evidently increased (both *P* < 0.001). PBD was found to prevent the deterioration of liver function. Within the PG group, Hb levels improved significantly (*P* < 0.001), while ALT, AST, TB, and DB decreased significantly (*P* < 0.001, *P* < 0.001, *P* < 0.001, and *P* < 0.001, respectively).

**Table 3:** A comparison of laboratory testing results between two time points (initial diagnosis and perioperative testing) within each group

Parameters	DG		Within DG			PG		Within PG		
	Initial	Perioperative	Cases with paired data available	Statistic	*P* value	Initial	Perioperative	Cases with paired data available	Statistic	*P* value
Hb (g/L)	126.13 ± 14.34	122.97 ± 14.71	124	*t* = 5.400	< 0.001	121.47 ± 17.51	114.70 ± 15.78	147	*t* = 6.352	< 0.001
WBC (10^9/L)	6.12 ± 1.84	5.98 ± 1.75	124	*t* = 1.463	0.146	6.45 ± 2.10	6.34 ± 2.00	147	*t* = 0.639	0.524
NEUT (%)	63.10 ± 9.36	64.23 ± 9.31	124	*t* = −1.789	0.076	66.94 ± 9.27	62.30 ± 9.37	146	*t* = 4.705	0.028
Plt (10^9/L)	252.23 ± 83.36	254.86 ± 85.68	124	*t* = −0.751	0.454	253.24 ± 87.03	243.80 ± 83.14	147	*t* = 1.764	< 0.001
ALB (g/L)	38.82 ± 4.09	38.17 ± 4.05	121	*t* = 2.435	0.016	37.43 ± 4.99	40.70 ± 5.22	147	*t* = −5.914	0.100
ALT (U/L)	200.21 ± 151.89	165.80 ± 140.32	122	*t* = 4.517	< 0.001	166.09 ± 152.17	78.52 ± 59.40	147	*t* = 7.656	< 0.001
AST (U/L)	133.94 ± 99.47	115.38 ± 93.63	122	*t* = 2.887	0.005	131.58 ± 122.80	54.60 ± 43.18	146	*t* = 7.882	0.001
TB (μmol/L)	140.99 ± 83.27	158.12 ± 95.11	122	*t* = −4.572	< 0.001	227.38 ± 126.49	83.03 ± 57.59	147	*t* = 14.399	< 0.001
DB (μmol/L)	116.11 ± 70.21	133.40 ± 79.21	121	*t* = −5.402	< 0.001	186.06 ± 103.03	71.49 ± 49.17	147	*t* = 13.904	< 0.001
GGT (U/L)	798.17 ± 602.69	739.93 ± 593.06	119	*t* = 3.228	0.002	586.88 ± 436.73	228.60 ± 181.69	145	*t* = 11.031	< 0.001
ALP (U/L)	434.24 ± 268.60	423.75 ± 262.36	122	*t* = 1.031	0.305	465.28 ± 363.83	230.40 ± 158.91	145	*t* = 9.484	< 0.001
Na (mmol/L)	139.83 ± 3.01	139.74 ± 3.16	119	*t* = 0.460	0.646	138.87 ± 3.16	139.78 ± 2.96	146	*t* = −3.279	< 0.001
PT (s)	11.35 ± 1.42	11.20 ± 1.22	117	*t* = 0.843	0.401	11.59 ± 1.51	11.65 ± 1.08	146	*t* = −0.402	0.682

*Hb* hemoglobin, *WBC* white blood cell, *NEUT* neutrophils, *Plt* platelet, *ALB* albumin, *ALT* alanine transminase-glutamic pyruvic transaminase, *AST* aspartate transminase-glutamic oxalo-acetic transaminase, *TB* total bilirubin, *DB* direct bilirubin, *GGT* γ-glutamyltransferase, *ALP* alkaline phosphatase, *Na* natrium, *PT* prothrombin time

A comparison of the laboratory test results between the two groups is presented in Table 4. At the time of initial diagnosis, the PG patients had worse anemia, higher percentage of neutrophils (NEUT %), hypoalbuminemia, and jaundice (*P* = 0.015, *P* = 0.001, *P* = 0.038, and *P* < 0.001, respectively). As to the perioperative laboratory findings, the PG patients’ condition reversed in terms of neutrophil percentage, hypoalbuminemia, and jaundice (*P* = 0.050, < 0.001, and < 0.001, respectively), but their anemia did not improve (*P* < 0.001).

**Table 4:** A comparison of laboratory testing results at two time points (initial diagnosis and perioperative testing) between the two groups

Characteristics	DG		PG		Initial data compared between groups		Perioperative data compared between groups	
	Initial	Perioperative	Initial	Perioperative	Statistic	*P* value	Statistic	*P* value
Hb (g/L)	125.85 ± 14.41	123.11 ± 14.74	121.17 ± 17.58	115.35 ± 15.93	*t* = 2.441	0.015	*t* = 4.193	< 0.001
WBC (10^9^/L)	6.11 ± 1.82	6.00 ± 1.76	6.55 ± 2.26	6.37 ± 2.04	*t* = −1.754	0.081	*t* = −1.670	0.096
NEUT (%)	63.15 ± 9.44	64.16 ± 9.31	67.11 ± 9.40	61.9 ± 9.51	*t* = −3.504	0.001	*t* = 1.972	0.050
Plt (10^9^/L)	250.5 ± 82.90	255.24 ± 85.44	255.6 ± 88.94	248.31 ± 84.76	*t* = −0.494	0.662	*t* = 0.679	0.498
ALB (g/L)	38.57 ± 4.16	38.21 ± 4.06	37.42 ± 4.96	40.75 ± 5.23	*t* = 2.085	0.038	*t* = −4.552	< 0.001
ALT (U/L)	198.78 ± 153.36	165.80 ± 140.32	166.10 ± 150.58	79.19 ± 62.58	*t* = 1.799	0.073	*t* = 6.340	< 0.001
AST (U/L)	134.50 ± 99.18	115.38 ± 93.63	131.63 ± 121.91	55.24 ± 44.08	*t* = 0.140	0.889	*t* = 6.530	< 0.001
TB (μmol/L)	140.84 ± 82.94	158.12 ± 95.11	225.1 ± 126.27	81.76 ± 56.74	*t* = −6.695	<0.001	*t* = 7.837	< 0.001
DB (μmol/L)	115.70 ± 69.55	132.52 ± 79.48	184.20 ± 102.96	70.36 ± 48.47	*t* = −6.601	<0.001	*t* = 7.598	< 0.001
GGT (U/L)	812.81 ± 627.21	736.18 ± 592.00	582.53 ± 434.59	224.00 ± 178.62	*t* = 3.501	0.001	*t* = 9.160	< 0.001
ALP (U/L)	430.06 ± 265.00	423.75 ± 262.36	465.36 ± 362.82	230.09 ± 157.57	*t* = −0.936	0.350	*t* = 7.195	< 0.001
Na (mmol/L)	139.74 ± 3.03	139.75 ± 3.15	138.88 ± 3.18	139.87 ± 2.96	*t* = 2.303	0.022	*t* = −0.327	0.728
PT (s)	11.53 ± 2.12	11.16 ± 1.21	11.569 ± 1.50	11.69 ± 1.09	*t* = −0.171	0.864	*t* = −3.824	< 0.001

### All postoperative outcomes and risk factors for postoperative complications

The postoperative outcomes are shown in Table 5. The overall rate of postoperative complications was 46.4% (134 patients out of 290), and complications occurred more frequently in the DG group (*P =* 0.029). The postoperative hemorrhage rate was significantly higher in the DG group (*P =* 0.038), whereas postoperative delayed gastric emptying was significantly more frequent in the PG group (*P =* 0.065). The rates of mortality and other complications were similar between the two groups. The location of obstruction was used to decide on the main surgical procedure, and each group was divided into a proximal-obstruction subgroup and distal-obstruction subgroup. The overall rate of postoperative complications in the combined proximal-obstruction group was 77.9%, and complications occurred more frequently in the proximal-obstruction DG subgroup (*P =* 0.038, Table 6). In the combined distal-obstruction group, the overall rate of postoperative complications was 50.8%, and PBD did not affect this rate (*P =* 0.249, Table 6).

**Table 5:** All postoperative complications in the study population

	Total (*n* = 290)	DG (*n* = 131)	PG (*n* = 159)	Statistic	*P* value
All complications	134	70	64	*χ*^2^ = 5.002	0.025
Intestinal fistula	23	10	13	*χ*^2^ = 0.029	0.865
Pancreatic fistula	20	10	10	*χ*^2^ = 0.202	0.653
Biliary fistula	1	0	1	-	-
Gastrointestinal anastomotic fistula	2	0	2	-	-
Abdominal liquid accumulation	31	16	15	*χ*^2^ = 0.581	0.446
Delayed gastric emptying	17	4	13	*χ*^2^ = 3.415	0.065
Hemorrhage	21	14	7	*χ*^2^ = 4.223	0.040
Hepatic failure	6	3	3	-	1.000
Reoperation	11	6	5	*χ*^2^ = 0.406	0.552
Incision complication	8	3	5	*χ*^2^ = 0.196	0.733
Mortality	6	3	3	-	1.000
Sepsis	78	41	37	*χ*^2^ = 2.354	0.125

**Table 6:** Postoperative outcomes in proximal- and distal-obstruction groups

	Total	DG	PG	Statistic	*P* value
Proximal obstruction (number of patients)	172	82	90	*χ*^2^ = 4.295	0.038
Complication(s) (number of patients)	134	70	64		
Distal obstruction	118	49	69	*χ*^2^ = 1.329	0.249
Complication(s)	60	28	32		

In univariate analysis, PBD was found to be associated with better postoperative outcomes (*P =* 0.025; Table 7). Perioperative Hb and perioperative TB were associated with postoperative complications (*P =* 0.027 and *P =* 0.016, respectively). Multivariate analysis indicated that perioperative TB is an independent risk factor for postoperative complications (Table 7).

**Table 7:** Univariate and multivariate analyses of factors possibly associated with postoperative outcomes in the study population (290 patients)

	Univariate analysis			Multivariate analysis		
	HR	95% CI	*P* value	HR	95% CI	*P* value
Age > 70	0.831	0.456‑1.515	0.545			
Gender	0.971	0.601‑1.570	0.906			
Tumor site	0.970	0.606‑1.553	0.900			
PBD	0.587	0.368‑0.937	**0.025**			0.163
Initial Hb	1.012	0.997‑1.027	0.111			
Perioperative Hb	1.017	1.002‑1.033	**0.027**			0.059
Initial WBC	0.953	0.849‑1.068	0.407			
Perioperative WBC	1.006	0.891‑1.137	0.922			
Initial TB	0.999	0.997‑1.001	0.516			
Perioperative TB	1.004	1.001‑1.007	**0.016**	1.004	1.001‑1.007	0.017
Initial GGT	1.000	1.000‑1.000	0.860			
Perioperative GGT	1.000	1.000‑1.001	0.893			
Initial Na	0.936	0.867‑1.011	**0.091**			0.184
Perioperative Na	0.990	0.916‑1.071	0.806			
Initial PT	0.962	0.841‑1.100	0.567			
Perioperative PT	0.915	0.746‑1.123	0.395			

*HR* hazard ratio, *CI* confidence interval, *GGT* gamma-glutamyl transferase

**Fig. 1:**
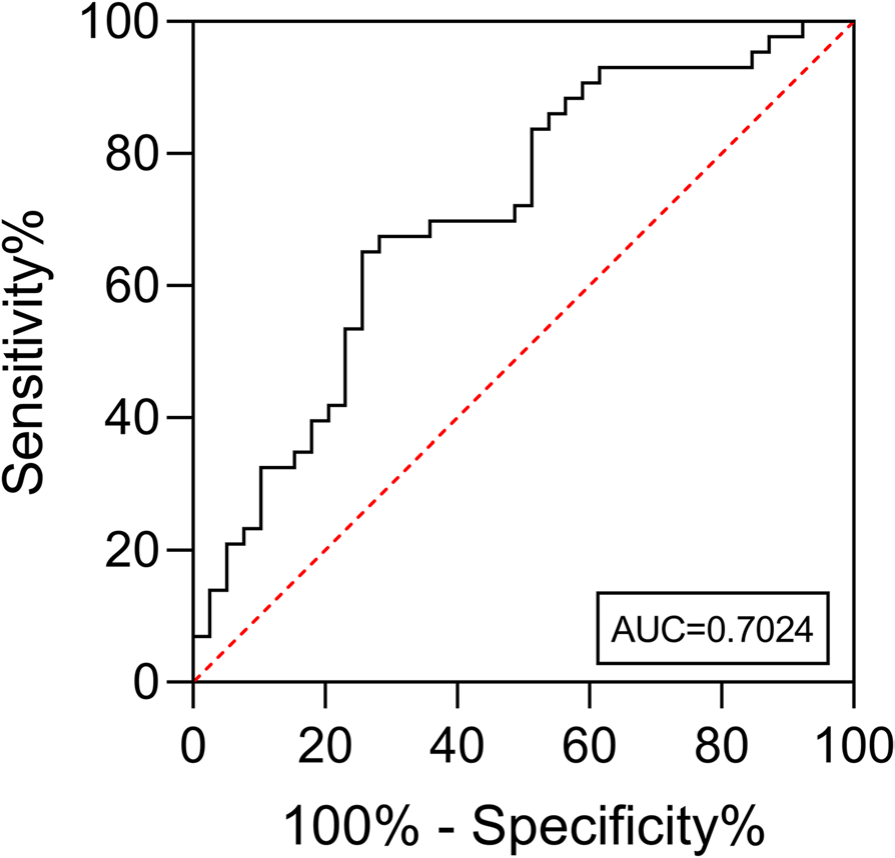
The ROC curve. This analysis shows that the best cutoff of preoperative TB for discriminating patients with complications from those without complications is 162 μmol/L (*P* = 0.002, sensitivity 62.8%, specificity 74.4%, AUC = 0.7024 [95% CI 0.588–0.816])

Preoperative TB concentration > 162 μmol/L distinguished patients with postoperative complication(s) from those without (Fig. 1, ROC curve analysis). For this cutoff, the area under the ROC curve was 0.7024 (95% confidence interval 0.588–0.816) (*P* = 0.002, sensitivity 62.8%, specificity 74.4%).

## Discussion

In this retrospective study, we analyzed the efficacy of PBD in patients with malignant obstructive jaundice. The incidence of patients with several different malignancies seemed puzzling. However, we want to emphasize that patients often do not undergo full medical examination at their first visit but receive preliminary treatment with limited data. Thus, we wanted to identify some easily accessible indicators that could help patients receive treatments as early as possible.

Obstructive jaundice is a life-threatening problem in patients with malignant tumors. Although the degree of hyperbilirubinemia is not necessarily related to tumor stage, hyperbilirubinemia interferes with many organ functions and limits treatment choices. Bilirubin, as a product of heme metabolism, is nearly nontoxic in its normal physiological range of concentrations, whereas hyperbilirubinemia is toxic requiring immediate attention. In particular, it has been associated with an increased risk of postoperative complications. PBD is a kind of “bridge therapy” before a radical surgical resection is attempted for malignant obstructive jaundice. There is no consensus on indications for PBD so far.

The retrospective data showed that PBD may reduce the overall rate of postoperative complications among patients with proximal obstructive jaundice. Perioperative TB turned out to be an independent risk factor for postoperative complications. Moreover, preoperative TB > 162 μmol/L distinguished patients with postoperative complications from those without.

PBD has had varied effects in different studies. Specifically, different PBD options have manifested various effects in different cancers. It must be emphasized that the data published so far came from diverse studies, where the patients had a small number of cancer types affecting the biliary tree. In 2010, van der Gaag et al. conducted a multicenter randomized controlled trial regarding the cancer of the pancreatic head and demonstrated that routine PBD increases the rate of complications. This conclusion was based on the bilirubin range of 40 to 250 μmol/L and was made about the cancer of the pancreatic head [12]. In 2016, a meta-analysis suggested that the PBD group has significantly fewer major adverse effects than the direct surgery group [11]. This conclusion was made about a series of tumors, e.g., cancers of the biliary tract, cancers of the head and neck, and cancers of the duodenum. On the other hand, the studies included in this meta-analysis took place between 1981 and 2011. Nearly simultaneously, another meta-analysis suggested that PBD worsens postoperative outcomes, increasing the prevalence rate of infectious complications, surgical site infections, and delayed gastric emptying [13]. Few studies have been conducted on multicenter randomized controlled trials for malignant obstructive jaundice [14]. In recent years, several PBD methods were simultaneously employed, and new drainage brackets are developed [15, 16]. The management of obstructive jaundice is complicated and involves not only surgeons and medical centers but also the entire healthcare system [17]. Hegel’s principle of “what exists is reasonable” is thought to be applicable worldwide, but we found a more accurate observation by examining the current methods in some clinical settings. For example, a Japanese retrospective study covered a 10-year clinical experience and revealed that PBD reduces postoperative morbidity and mortality in the whole country regardless of the PBD type [18]. This finding suggests that a beneficial modality can also be found at our medical center. During the study period, we applied a mature surgical technology and advanced equipment, and the observed prevalence of postoperative complications is not consistent with other hospitals and countries.

Our results show a clear difference made by PBD among patients with malignant obstructive jaundice. This report is the first retrospective study with the latest technology to show benefits of PBD for patients with malignant obstructive jaundice in China. Accurate preoperative diagnosis of biliary tract tumors is difficult, but the estimation of an obstruction site is easier. On the basis of our study, we believe that patients with proximal obstruction should consider PBD more often. Due to the limited number of cases in the recent 2 years under study, we did not obtain strong evidence that PBD affects postoperative complications of patients with distal obstruction.

This study has certain limitations. First, this study was retrospective, so selection bias potentially occurred. Although the best way to reduce or eliminate selection bias is a randomized controlled trial, such a trial is not feasible for patients with malignant obstructive jaundice. Second, the study involved a small sample size from only one hospital in China with predominantly Chinese patients. Medical treatment level differs widely among regions of China, so data in this study could not be widely representative.

## Conclusion

Our study indicates that PBD may reduce the overall rate of postoperative complications in patients with proximal malignant obstructive jaundice. Perioperative TB is an independent risk factor for postoperative complications and supports the decision to recommend preoperative PBD when preoperative TB is > 162 μmol/L.

## Data Availability

The data used to support the findings of this study are available from the corresponding author upon request.
